# Predicting disease progression in multiple sclerosis with clinically accessible information and technology

**DOI:** 10.1007/s00415-026-13802-4

**Published:** 2026-04-19

**Authors:** Tom A. N. Fuchs, Menno M. Schoonheim, Eva M. M. Strijbis, Julia R. Jelgerhuis, Dana Horakova, Eva K. Havrdova, Tomas Uher, Robert Zivadinov, Serkan Ozakbas, Marc Girard, Raed Alroughani, Pierre Grammond, Alessandra Lugaresi, Valentina Tomassini, Tomas Kalincik, Izanne Roos, Oliver Gerlach, Anneke van der Walt, Samia J. Khoury, Vincent van Pesch, Andrea Surcinelli, Matteo Foschi, Maria Jose Sa, Emanuelle D’amico, Jens Kuhle, Elisabetta Cartechini, Davide Maimone, Rana Karabudak, Aysun Soysal, Daniele Spitaleri, Guy Laureys, Bruce Taylor, Marie D’hooghe, Radek Ampapa, Tamara Castillo-Triviño, Ayse Altintas, Orla Gray, Riadh Gouider, Jose E. Meca-Lallana, Allan G. Kermode, Marzena Fabis-Pedrini, William M. Carroll, Koen de Gans, Jose Luis Sanchez-Menoyo, Masoud Etemadifar, Abdullah Al-Asmi, Pamela McCombe, Mihaela Simu, Mehmet Fatih Yetkin, Talal Al-Harbi, Tunde Csepany, Patrice Lalive, Todd A. Hardy, Sudarshini Ramanathan, Barbara Willekens, Angel Perez Sempere, Simón Cárdenas-Robledo, Mario Habek, Bhim Singhal, Nikolaos Grigoriadis, Magdolna Simo, Vahid Shaygannejad, Yolanda Blanco, Eduardo Aguera-Morales, Justin Garber, Claudio Solaro, Neil Shuey, Dheeraj Khurana, Danny Decoo, Abdorreza Naser Moghadasi, Katherine Buzzard, Olga Skibina, Nevin John, Thor Petersen, Bianca Weinstock-Guttman

**Affiliations:** 1 MS Center Amsterdam, Vrije Universiteit Amsterdam, Amsterdam UMC, Location VUmc, De Boelelaan 1108, 1081 HZ Amsterdam, The Netherlands; 2 Charles University in Prague and General University Hospital, Prague, Czech Republic; 3 Jacobs School of Medicine and Biomedical Sciences, Buffalo, USA; 4 Izmir University of Economics, Medical Point Hospital, Izmir, Turkey; 5 CHUM and Universite de Montreal, Montreal, Canada; 6 Amiri Hospital, Sharq, Kuwait; 7 CISSS Chaudière-Appalache, Levis, Canada; 8 Università di Bologna, Bologna, Italia; 9 University G. d’Annunzio of Chieti-Pescara, Chieti, Italy; 10 Department of Neurology, Neroimmunology Centre, Royal Melbourne Hospital, Melbourne, Australia; 11 Zuyderland Medical Center, Sittard-Geleen, The Netherlands; 12 Department of Neurology, The Alfred Hospital, Melbourne, Australia; 13 American University of Beirut Medical Center, Beirut, Lebanon; 14 Cliniques Universitaires Saint-Luc, Brussels, Belgium; 15 S. Maria Delle Croci Hospital, AUSL Romagna, Ravenna, Italy; 16 Centro Hospitalar Universitario de Sao Joao, Porto, Portugal; 17 Medical and Surgical Sciences, Universita di Foggia, Foggia, Italy; 18 University Hospital Basel, Basel, Switzerland; 19 Neurology Unit, AST Macerata, Macerata, Italy; 20 Azienda Ospedaliera per l’Emergenza Cannizzaro, Catania, Italy; 21 Yeditepe University, Istanbul, Turkey; 22 Bakirkoy Education and Research Hospital for Psychiatric and Neurological Diseases, Istanbul, Turkey; 23 Azienda Ospedaliera di rilievo Nazionale San Giuseppe Moscati Avellino, Avellino, Italy; 24 Universitary Hospital Ghent, Ghent, Belgium; 25 Royal Hobart Hospital, Hobart, Australia; 26 National MS Centrum, Melsbroek, Belgium; 27 Nemocnice Jihlava, Jihlava, Czech Republic; 28 Hospital Universitario Donostia and IIS Biodonostia, San Sebastián, Spain; 29 Koc University, Istanbul, Turkey; 30 South Eastern HSC Trust, Belfast, UK; 31 Razi University Hospital, Tunis, Tunisia; 32 Virgen de la Arrixaca Clinical University Hospital. IMIB-Arrixaca, Murcia, Spain; 33 Perron Institute for Neurological and Translational Science, The University of Western Australia, Perth, Australia; 34 Groene Hart Ziekenhuis, Gouda, The Netherlands; 35 Galdakao-Usansolo University Hospital, Osakidetza-Basque Health Service, Galdakao, Spain; 36 MS Institute, Osfahan, Iran; 37 College of Medicine and Health Sciences and Sultan, Qaboos University Hospital, Sultan Qaboos University, Al-Khodh, Oman; 38 Royal Brisbane and Women’s Hospital, Brisbane, Australia; 39 Department of Neuroscience Clinic of Neurology, University of Medicine and Pharmacy Victor Babes Timisoara, Timisoara, Romania; 40 Department of Neurology, Erciyes University, Kayseri, Turkey; 41 King Fahad Specialist Hospital-Dammam, Dammam, Saudi Arabia; 42 Department of Neurology, University of Debrecen, Debrecen, Hungary; 43 Faculty of Medicine, Geneva University Hospitals, Geneva, Switzerland; 44 Concord Repatriation General Hospital, University of Sydney, Sydney, Australia; 45 Department of Neurology, Antwerp University Hospital, Edegem, Belgium; 46 Hospital General Universitario de Alicante, Alicante, Spain; 47 Hospital Universitario Nacional de Colombia, Bogota, Colombia; 48 University Hospital Center Zagreb, Zagreb, Croatia; 49 Bombay Hospital Institute of Medical Sciences, Mumbai, India; 50 AHEPA University Hospital, Thessaloniki, Greece; 51 Semmelweis University Budapest, Budapest, Hungary; 52 Isfahan University of Medical Sciences, Isfahan, Iran; 53 Hospital Clinic de Barcelona, Barcelona, Spain; 54 University of Cordoba, Cordoba, Spain; 55 Department of Neurology, Westmead Hospital, Sydney, Australia; 56 Department of Neurology, Galliera Hospital, Genoa, Italy; 57 Vincent’s Hospital, Fitzroy, ,Melbourne, Australia; 58 Department of Neurology, Postgraduate Institute of Medical Education and Research, Chandigarh, India; 59 Alma Ziekenhuis, Sijsele, Damme, Belgium; 60 Multiple Sclerosis Research Center, Neuroscience Institute, Tehran University of Medical Sciences, Tehran, Iran; 61 Department of Neurosciences, Box Hill Hospital, Box Hill, Melbourne, Australia; 62 Department of Neurology, Monash University, Clayton, Australia; 63 Aarhus University Hospital, Arhus C, Denmark

**Keywords:** Multiple sclerosis, Decision support tools, Prediction, Clinical, Disease progression, Secondary progressive multiple sclerosis

## Abstract

**Background:**

Predicting disease progression at the individual level is essential for personalized medicine. We previously developed machine-learning tools to estimate 5-year progression risk in people with multiple sclerosis (PwMS). Such models should account for disease-modifying therapy (DMT) and objective outcome definitions.

**Methods:**

In a retrospective multicenter case–control study, we evaluated adults with relapsing–remitting multiple sclerosis (RRMS) at baseline. Using machine-learning, we developed two complementary tools for individualized 5-year risk estimation: DAAE-M, optimized for transparency, software-neutral use, and mitigation of indication bias, and ELIE, optimized for dynamic landmark-based modeling, complex treatment histories, and mitigation of immortal-time bias. Disease progression was defined using both a clinical outcome (RRMS-to-progressive MS) and an objective outcome (late-stage confirmed progression independent of relapse activity).

**Results:**

Among 34,510 people with RRMS (72.6% female, mean age = 37.1, mean disease duration = 5.8), 9.8% and 21% met clinical and objective progression criteria, respectively, over five years. Both models demonstrated good calibration across risk-groups (Brier scores 0.06–0.16). DAAE-M provided patient-level risk estimates with monotonic risk escalation across risk-groups for clinical (3.1%/11.2%/22.6%/33.0%) and objective (8.4%/14.5%/23.3%/38.8%) progression. For DAAE-M, high-efficacy DMT was associated with approximately half the progression risk compared with low-efficacy DMT (risk-ratios: 0.42–0.59; *p* < 0.01). ELIE also showed good calibration across risk deciles with increasing incidence for both clinical (0.3%/1.2%/1.7%/2.5%/3.7%/5.5%/7.2%/10.2%/14.3%/21.5%) and objective (0.9%/1.6%/2.5%/4.0%/5.8%/7.8%/10.2%/15.3%/20.9%/32.5%) outcomes.

**Conclusion:**

We developed two well-calibrated machine-learning-based tools for individualized 5-year prediction of clinically- and objectively-defined MS progression, each with distinct strengths in usability, bias handling, and treatment modeling. These findings support future tool use in personalized risk stratification and secondary prevention.

**Supplementary Information:**

The online version contains supplementary material available at 10.1007/s00415-026-13802-4.

## Introduction

Predicting disease progression is critical for decision support and personalized patient care—such as through identification of high-risk patients for secondary prevention and restorative treatment [1]. Consequently, numerous investigations have identified predictors of disease progression in people with multiple sclerosis (PwMS) [2].

With this background, we previously aimed to translate this science toward clinical practice by developing the DAAE score, a decision support tool for estimating individual patient risk of disease progression over 5 years using clinically accessible information and technology [3]. While DAAE score development employed machine learning techniques, its user-end functionality was purposefully designed to be transparent, software neutral, and easy to use with clinically available information and technology. Accordingly, the DAAE score incorporates four factors: Disease duration, Age, Age at disease onset, and the Expanded Disability Status Scale (EDSS), each weighted differently, offering a 0 to 12-point scale for patient risk stratification.

In line with WHO guidance emphasizing transparency, explainability, and intelligibility for AI-enabled clinical decision-making [4], we prioritized an explicit, user-facing scoring system that clinicians can compute rapidly and audit conceptually, rather than an opaque ‘black-box’ predictor. To facilitate implementation within heterogeneous health ecosystems, we designed the prognostic tool as a software-neutral scoring algorithm with lookup tables, aligning with WHO’s SMART Guidelines approach to standards-based, interoperable digital decision support components [5].

To ensure generalizability of risk estimates, it is important that performance is evaluated across clinical centers and heterogeneous populations using clinical [6, 7], and objective [8] definitions of disease progression. Additionally, such a tool has the greatest clinical benefit if it can help physicians make treatment decisions. It is therefore important to incorporate disease-modifying therapy (DMT) treatment effects and to establish whether risk estimates meaningfully vary according to therapy [9].

To improve the potential to support personalized patient care, this study had the following aims: using machine-learning, we aimed to develop two complementary tools for individualized 5-year risk predictions: The first, DAAE-M (Disease duration, Age, Age of onset, EDSS, disease Modifying therapy), was optimized according to guidelines for clinical interoperability and for mitigation of indication bias. The second for comparison, ELIE (Empirical Landmark-based Individualized Estimation of MS progression risk), was optimized for dynamic landmark modeling, complex treatment histories, and mitigation of immortal-time bias. Disease progression was defined using both a clinical outcome (RRMS-to-progressive MS) and an objective outcome (Lorscheider criteria, late-stage confirmed progression independent of relapse activity).

For both models, we aimed to:

1. Integrate DMT information: incorporate risk predictions depending on DMT use.
2. Multi-center validation: validate risk-stratification performance in a large multi-center clinical dataset
3. Validate against clinically defined disease progression
4. Validate against objectively defined disease progression

We hypothesized that (1) high-efficacy therapy would be associated with lower risk, (2) risk predictions would be well-calibrated to actual event proportions, (3) risk of transition would escalate across stratified risk groups for clinically defined and objectively defined disease progression.

## Methods

## Study design and participant selection

A multi-center longitudinal study was conducted using participant data from the MSBase registry, collected from 1964 to 2023. All participants provided written informed consent. Ethics approval for the MSBase registry was granted by the Alfred Health Human Research and Ethics Committee and the local ethics committees of all the participating centers. This study followed the Strengthening the Reporting of Observational Studies in Epidemiology (STROBE) reporting guidelines [10]. The criteria for patient inclusion were: (A) relapsing–remitting multiple sclerosis (RRMS) at baseline, (B) no other neurological disorder, (C) age 18 or older, (D) two or more clinical evaluations, (E) > 3 years longitudinal clinical follow-up [target of 5 years (± 2)], (F) baseline neurologic testing of disability using the EDSS, (G) data not previously used in DAAE score development. ‘Baseline’ was defined as the point of contact at year zero of the target 5-year observation window.

### Measures and outcome

A previous predictive algorithm was applied as described [3]. Briefly, four clinical factors (Disease duration, age, age at disease onset, EDSS) are considered, each with different weighting, to generate a score from 0 to 12 points. Then, PwMS are stratified into risk groups according to their score (very-low = 0–2, low = 3–7, medium = 8–9, high ≥ 10) and associated risk of disease progression. Original development of this employed machine-learning techniques, training and testing, and four key development stages: (1) feature identification with systematic literature review, (2) feature ordinalization using generalized additive modeling (GAM), (3) feature selection with least-absolute-shrinkage and selection operator regression (LASSO), (4) and feature weighting using training and testing paradigms with GAM and LASSO coefficients. See Fuchs et al. 2024 [3] for further details. Missing values were handled using multiple imputation with chained equations [11].

Disease progression was defined both clinically and objectively:

*Clinical disease progression*. First, to ensure tool validation was harmonized with real-world use, disease progression was defined according to neurologists’ judgment of transition to secondary progressive multiple sclerosis (SPMS). This outcome was chosen because people with SPMS accumulate disability more rapidly, do not respond to most disease modifying therapies, and benefit less from restorative rehabilitation [12–14]

*Objective disease progression*. We also defined disease progression objectively with regard to late-stage confirmed progression independent of relapse using the MSBase-Lorscheider criteria [8] (all criteria met):

- EDSS ≥ 4.0 overall, pyramidal functional subsystem score ≥ 2
- EDSS increase ≥ 1.0 in patients with EDSS ≤ 5.5 or EDSS increase ≥ 0.5 in patients with EDSS ≥ 6
- Disability worsening was confirmed stable after three months in EDSS and in leading functional subsystem
- No relapses were detected between the event date and the preceding clinical evaluation.

#### DMT efficacy scheme

High: rituximab, ocrelizumab, mitoxantrone, alemtuzumab, natalizumab, ofatumumab.

Low: interferon-beta, glatiramer acetate, teriflunomide, dimethyl fumarate.

Intermediate: fingolimod, siponimod, daclizumab, laquinimod, cladribine.

## Predictive model optimized for transparency, interoperability, and mitigation of indication bias

### Integrate DMT information

For this model, DAAE-M, user-functionality and clinical translational potential were optimized in the design of the final models in accordance with WHO guidelines [4, 5], with a focus on transparency, interoperability, and software-neutral design practices.

We hypothesized that risk of clinical disease progression would be lower for PwMS receiving high-efficacy DMT relative to those receiving low-efficacy DMT, and therefore aimed to integrate these risk differences. For these analyses, we categorized PwMS as either receiving low-efficacy or high-efficacy DMT. This was done in two ways, accounting for majority use of DMT over the observation time-horizon and for baseline DMT.

*Majority DMT use*: defined depending on which therapies were received over the majority (> 50%) of the 5-year observation period. After this, PwMS were considered to have switched therapy classes if they also received another class of DMT for > 25% of the observation period. Therapy efficacy was defined according to previously established categories [9, 15], and is described the DMT efficacy scheme section, above.

*Baseline DMT*: defined according to the therapy being used at baseline.

For direct comparison between these groups, we corrected for indication bias [16]. In this context, indication bias is the tendency for high-risk patients to receive high-efficacy therapy and for stable patients to receive no or low-efficacy therapy. To mitigate the effects of this bias, we used propensity-score matching between PwMS receiving high- versus low-efficacy DMT. Propensity scores were estimated using a logistic regression model, with the following baseline covariates: age, sex, disease duration, and EDSS. Matching was completed using nearest-neighbor 1:1 matching without replacement. Then, the DAAE score was applied to establish proportions of PwMS with disease progression in PwMS receiving high-efficacy versus low-efficacy DMT for each of the risk groups. This analysis approach was then repeated for PwMS receiving no treatment.

#### Statistical analyses

Proportions of events within each treatment group were compared to the low-efficacy DMT group using z-tests for proportions. Due to limitations in sample-size, these analyses excluded PwMS receiving intermediate-efficacy DMT.

### Multi-center validation

#### Clinically defined disease progression

For primary validation, we completed calibration analyses, comparing risk predictions to actual event proportions per stratified risk group. Additionally, although the tool was developed to stratify PwMS according to risk (percent) of disease progression over 5 years, we also evaluated discriminative prediction performance for comparison with other models and tools. For this secondary validation, we evaluated the area-under-receiver-operating-curve (AUROC) and percent prediction accuracy with bootstrapped 95% confidence intervals. To address class imbalance in discriminative predictions, we employed the synthetic minority oversampling technique combined with Tomek links (SMOTE + TK, k = 10) [17].

#### Objectively defined disease progression

Here, we aimed to establish the risk of disease progression according to objective disease progression criteria using the MSBase-Lorscheider [8]. In short, the MSBase-Lorscheider criteria provides a standardized approach for defining SPMS and late-stage confirmed disease progression independent of relapse by applying a disability cutoff and evidence of relapse-free disability worsening sustained over three months. For details, see Measures and Outcome.

For this analysis, the sample was reduced to PwMS that met initial inclusion requirements and these additional criteria needed for calculating the Lorscheider-MSBase criteria: (H) clinical relapse reporting (yes/no, date), (I) availability of all EDSS functional subsystem data, (J) > 3 months between evaluations, and (K) RRMS at baseline according to objective criteria. Finally, because MSBase-Lorscheider criteria are less conservative than clinical judgment [8], DAAE score risk-group stratification cut-offs were adapted for this outcome.

#### Statistical analysis

To compare predicted vs actual risk estimates, 95% confidence intervals and z-tests for proportions were applied. These were also applied to evaluate the distinctness of event proportions across each of the DAAE-M score-stratified risk groups.

## Predictive model optimizing for dynamic estimation of risk and mitigation of immortal time bias

To optimize for dynamic predictions, incorporate more complex treatment histories, and mitigate immortal time bias [18] in predictive modeling with retrospective data, we developed a dynamic landmark-based prediction model, ELIE, to estimate individual patient risk of disease progression. Disease progression was defined clinically [SPMS] and objectively [Lorschieder criteria], as above, within a 5-year fixed prediction horizon after the landmark visit. Each eligible clinical visit served as a landmark time point, generating a visit-level prediction dataset in which patients could contribute multiple observations over follow-up. Only visits occurring prior to the outcomes were included. Landmarks were censored if follow-up was insufficient to determine outcome status.

Predictors included demographic and clinical variables measured at the landmark visit (age, disease duration, EDSS, age at onset, time since baseline), as well as a categorical summary of disease-modifying therapy (DMT) exposure up to the landmark. DMT history was derived from longitudinal treatment records and summarized as a time-varying categorical variable reflecting predominant exposure and treatment switching patterns prior to each landmark.

For each landmark, treatment intervals were intersected with the observation window (baseline → landmark date). The proportion of time exposed to each efficacy class was calculated relative to the total observed time. DMTs were categorized into three efficacy classes (low-, intermediate-, and high-efficacy), consistent with prior classification schemes.

A majority exposure class was defined as ≥ 50% of observed time spent on a given efficacy class. A “switcher” flag was defined when ≥ 50% of observed time was spent on treatment and ≥ 25% of treated time was spent on a non-dominant efficacy class. In addition, transitions between efficacy classes were enumerated in chronological order to detect escalation, de-escalation, initiation, discontinuation, and bidirectional switching. Each landmark was assigned to one mutually exclusive treatment history category as follows:

Never treated, no DMT exposure prior to the landmark (majority class = None; no switching); Stable low-efficacy, ≥ 50% of time on low-efficacy DMT with no detected switching; Stable intermediate-efficacy, ≥ 50% of time on intermediate-efficacy DMT with no detected switching; Stable high-efficacy, ≥ 50% of time on high-efficacy DMT with no detected switching; Switcher escalation, at least one transition from lower- to higher-efficacy therapy (e.g., low → high), without reverse transition; Switcher de-escalation, at least one transition from higher- to lower-efficacy therapy (e.g., high → low), without reverse transition; Switcher bidirectional, both escalation and de-escalation occurred prior to the landmark; Switcher initiation, transition from no therapy to treated state (None → any efficacy), without discontinuation; Switcher discontinuation, transition from treated to untreated (any efficacy → None), without re-initiation; Switcher complex, switching patterns not captured by the above rules (e.g., multiple transitions not fitting a single directional pattern).

To prevent information leakage, data were split into training and test sets (80:20) at the patient level, and cross-validation was likewise grouped by patient. Models were fitted using weighted LASSO regression (glmnet), with class weights to address outcome imbalance. Preprocessing, including imputation, dummy encoding of categorical predictors, and standardization of continuous variables, was performed using a fixed recipe learned on the training data. Within the training set, the LASSO penalty parameter was optimized using patient-grouped cross-validation under class-weighted loss. Finally, resulting model performance was evaluated in the independent test set using calibration (primary validation) and discrimination (secondary validation) metrics, as described in previous sections.

### Statistical analyses

To compare predicted vs actual risk estimates, 95% confidence intervals and z-tests for proportions were applied. Due to the high degree of comparisons (10 deciles per outcome), p-values were Bonferroni-corrected.

## Study funding

This work was supported by the European Committee for Treatment and Research in Multiple Sclerosis (ECTRIMS), financially supporting the salary of the principal author Dr. Tom A.N. Fuchs.

## Result

### Descriptive statistics

Of the 114,476 patient records available within the MSBase registry, 34,510 PwMS (100% RRMS at baseline) met inclusion criteria for the present study, with 72.6% being female (*n* = 25,071). For further details, see the CONSORT diagram in Supplemental. At baseline, the mean (SD) age was 37.1 (10.8) years, and the disease duration was 5.8 (7.3) years. The baseline median [IQR] EDSS score was 2.0 (1.0–3.0). The PwMS were observed for a mean (SD) of 4.8 (1.2) years. For further details, see Table 1.

**Table 1:** Sample characteristics

RRMS (*n*, %)	34,510, 100%
Female (*n*, %)	25,071, 72.6%
Age in years (mean, SD)	37.1, 10.8
Disease duration in years (mean, SD)	5.8, 7.3
Majority DMT use (> 50%) by efficacy*	
Low efficacy (*n*, %)	14,691, 42.6%
High efficacy (*n*, %)	2482, 7.2%
Switch (*n*, %)	4133, 12.2%
No therapy (*n*, %)	11,179, 32.4%
Baseline DMT use by efficacy	
Low efficacy (*n*, %)	19,041, 55.2%
High efficacy (*n*, %)	2596, 7.5%
No therapy (*n*, %)	12,873, 37.3%
Majority DMT use (> 50%) by name*	
Cladribine	110, 0.32%
Fingolimod	2417, 7.0%
Interferon	9621, 27.88%
Teriflunomide	787, 2.28%
Alemtuzumab	57, 0.17%
Daclizumab	30, 0.09%
Laquinimod	3, 0.01%
Mitoxantrone	43, 0.12%
Natalizumab	1823, 5.28%
Ocrelizumab	681, 1.97%
Ofatumumab	5, 0.01%
Rituximab	208, 0.6%
Siponimod	4, 0.01%
EDSS (median, IQR)	2.0, 1.0–3.0
Transition to SPMS	3391, 9.8%
Years observed	4.8, 1.2

*RRMS* relapsing–remitting multiple sclerosis, *SPMS* secondary progressive multiple sclerosis, *SD* standard deviation, *DMT* disease modifying therapy, *EDSS* Expanded Disability Status Scale, *IQR* inter-quartile range

*Majority DMT use was considered if receiving therapy for > 50% of the observation period. See methods for additional details

## Predictive model optimized for transparency, interoperability, and mitigation of indication bias (DAAE-M)

### Majority DMT use

Here, we aimed to account for time-varying DMT use in a risk-stratification system for predicting risk of disease progression in individual patients over 5 years—optimized for clinical usability, software interoperability, transparency, and mitigation of indication bias. This new scoring system is -referred to as the DAAE-M score, named for the predictors included in the final model: Disease duration, Age, Age at disease onset, EDSS, and disease Modifying therapy. Over the 5-year time-horizon, 42.6% (14,691) of the PwMS were receiving low-efficacy DMT and 7.2% (2,482) were receiving high-efficacy DMT for the majority (> 50%) of the observation period.

In propensity-score-matched majority low-efficacy (*n* = 2482) and high-efficacy (*n* = 2482) DMT groups, the proportion of PwMS exhibiting clinically-defined disease progression escalated concordantly with stratified risk groups (very-low, low, medium, high). However, the risk was nearly half for PwMS receiving high-efficacy DMT in each stratified risk group (very-low = 1.8%, low = 6.6%, medium = 13.5%, high = 15.2%) relative to PwMS receiving low-efficacy DMT (very-low = 3.2%, low = 10.3%, medium = 18.4%, high = 40.6%). This reduced risk was significant in particular for those in the very low- (risk-ratio(RR) = 0.54 [95% CI 0.30–0.96], *p* = 0.038), low (RR = 0.64 [95% CI 0.48–0.85], *p* = 0.002), and high-risk groups (RR = 0.37 [95% CI 0.25–0.55], *p* < 0.001), and non-significant for the medium-risk group (RR = 0.73 [95% CI 0.49–1.08], *p* = 0.114).

**Fig. 1:**
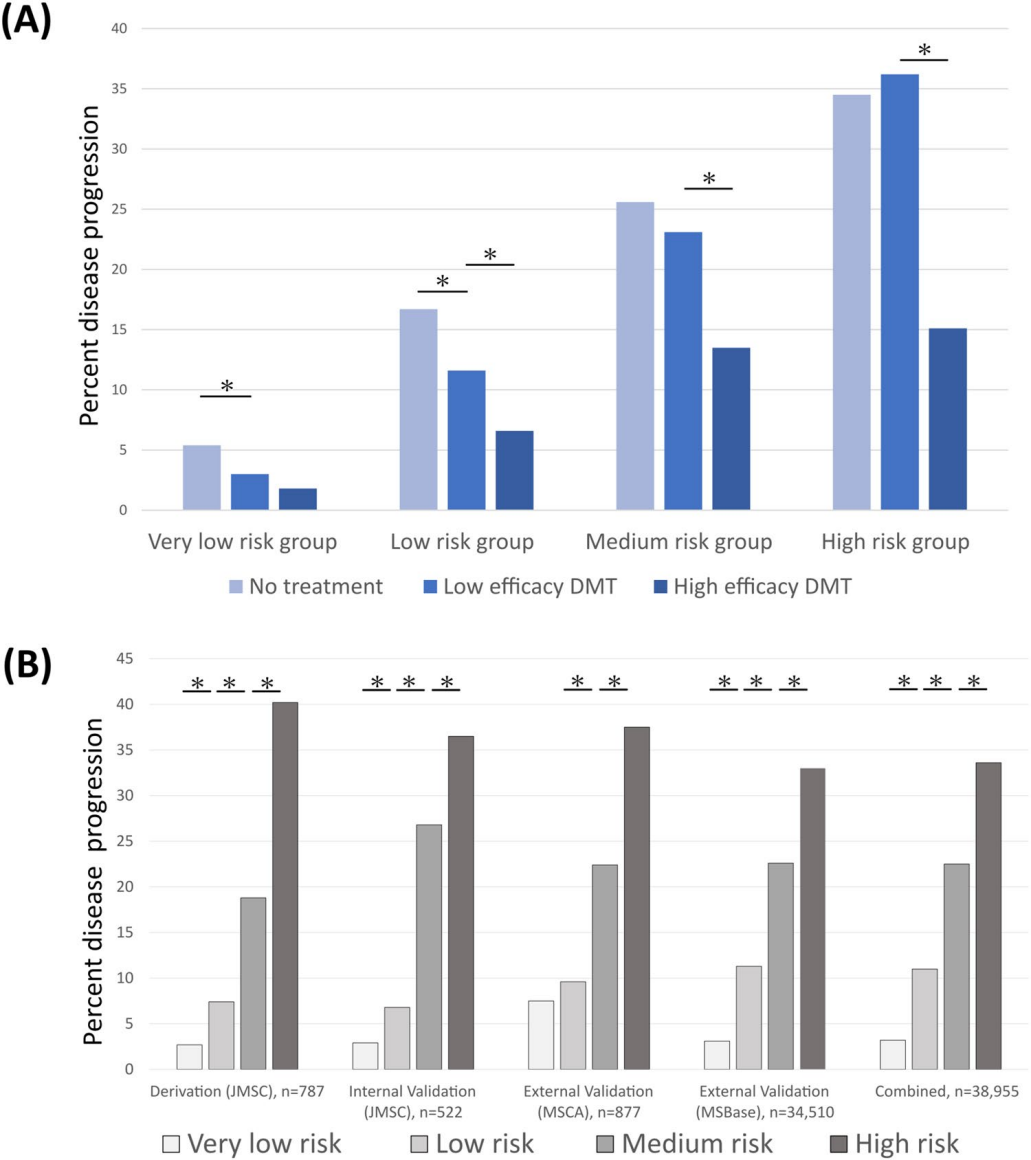
Validation and DMT integration (DAAE-M score). DMT integration (**A**) Risk of clinically-defined disease progression (transition to SPMS) in propensity-score-matched patient groups receiving low-efficacy DMT, high-efficacy DMT, or no treatment over the 5-year observation window. Event proportions (**B**). The proportion of patients with clinical disease progression across DAAE score-stratified risk groups (very-low, low, medium, and high) for the development (JMSC), internal validation (JMSC), external validation (MSCA), and the present multi-center external validation (MSBase). *p < 0.05 using a z-test for proportions. Abbreviations: *DMT* disease-modifying therapy, *JMSC* Jacobs Multiple Sclerosis Center, *MSCA* Multiple Sclerosis Center Amsterdam, *CUMC* Charles University Medical Center

Proportions of PwMS exhibiting clinical disease progression in the propensity-score-matched no-treatment group (*n* = 2482) also escalated concordantly with stratified risk groups (very-low = 5.4%, low = 15.7%, medium = 25.6%, high = 34.5%). Additionally, relative to the low-efficacy treatment group, risk was significantly higher, in particular for the very-low- (RR = 1.58 [95% CI 1.03–2.44], *p* = 0.003), low- group (RR = 1.62 [95%CI 1.31–2.03], *p* < 0.001), and medium-risk group (RR = 1.46 [95% CI 1.03–2.06], *p* = 0.032). This difference was non-significantly different for the high-risk group (RR = 0.86 [95% CI 0.64–1.16], *p* = 0.323). For details, see Table 2 and Fig. 1A.

**Table 2:** Clinical disease progression risk stratification, accounting for DMT use* over the 5-year observation window (DAAE-M)

	DAAE-M score	0–2	3–7	8–9	≥ 10
	Risk group	Very low	Low	Medium	High
Clinical disease progression % (95% CI)					
Unspecified therapy	–	3.1% (2.9–3.4)	11.2% (10.7–11.8)	22.6% (21.1–24.1)	33.4% (31.0–35.1)
Majority DMT*	Low-efficacy DMT	3.3% (2.3–4.6)	10.3% (8.5–12.3)	18.4% (13.8–23.8)	40.6% (33.9–47.5)
	High-efficacy DMT	1.8% (1.0–2.8)	6.6% (5.2–8.2)	13.5% (9.8–17.9)	15.2% (10.1–21.5)
	No DMT	5.2% (3.9–6.8)	16.7% (14.6–19.0)	26.9% (21.0–33.5)	35% (26.5–44.2)
Baseline DMT	Low-efficacy DMT	3.8% (2.6–5.3)	12.3% (10.4–14.5)	24.4% (20.2–29.1)	35.5% (30.0–41.2)
	High-efficacy DMT	2.1% (1.2–3.3)	8.7% (7.2–10.5)	18.8% (14.9–23.0)	25.4% (19.7–31.7)
	No DMT	3.6% (2.5–5.1)	12.1% (10.3–14.2)	23.0% (18.5–28.0)	29.3% (23.9–35.1)

*CI* confidence interval, *DMT* disease-modifying therapy

*DMT history is defined according to the majority (> 50%) use of the five-year observation period. Unspecified therapy relates to data from the full study sample, regardless of DMT history

**Insufficient data was available for risk stratification in people who switched DMT class over the observation period

Risk-stratification analyses were not completed for the group that switched DMT classes for the following reasons: Small representation of study sample (< 1% per paradigm); Sample distribution largely without representation in the medium- (*n* = 1) and high-risk (*n* = 0) groups; Heterogeneity of treatment histories in this sample in which 0.6% (n = 245) represent treatment escalation (low-efficacy to high-efficacy) and 0.5% (*n* = 213) represent de-escalation (high-efficacy to low-efficacy). For details on sample sizes across risk groups, see Supplementary Table A.

### Baseline DMT use

Here, we aimed to account for baseline DMT use. At baseline, 55.2% (n = 19,041) of the PwMS were receiving low-efficacy DMT and 7.5% (*n* = 2596) were receiving high-efficacy DMT.

In propensity-score-matched low-efficacy (*n* = 2596) and high-efficacy (*n* = 2596) DMT groups, the proportion of PwMS exhibiting clinically-defined disease progression escalated concordantly with stratified risk groups (very-low, low, medium, high). Proportions of PwMS exhibiting clinical disease progression in the propensity-score-matched no-treatment group (*n* = 2596) also escalated concordantly with stratified risk groups (very-low, low, medium, high). Risk was similar regardless of baseline treatment. For details, see Table 2.

### Multi-center validation (DAAE-M)

#### Clinical disease progression (transition to SPMS)

**Fig. 2:**
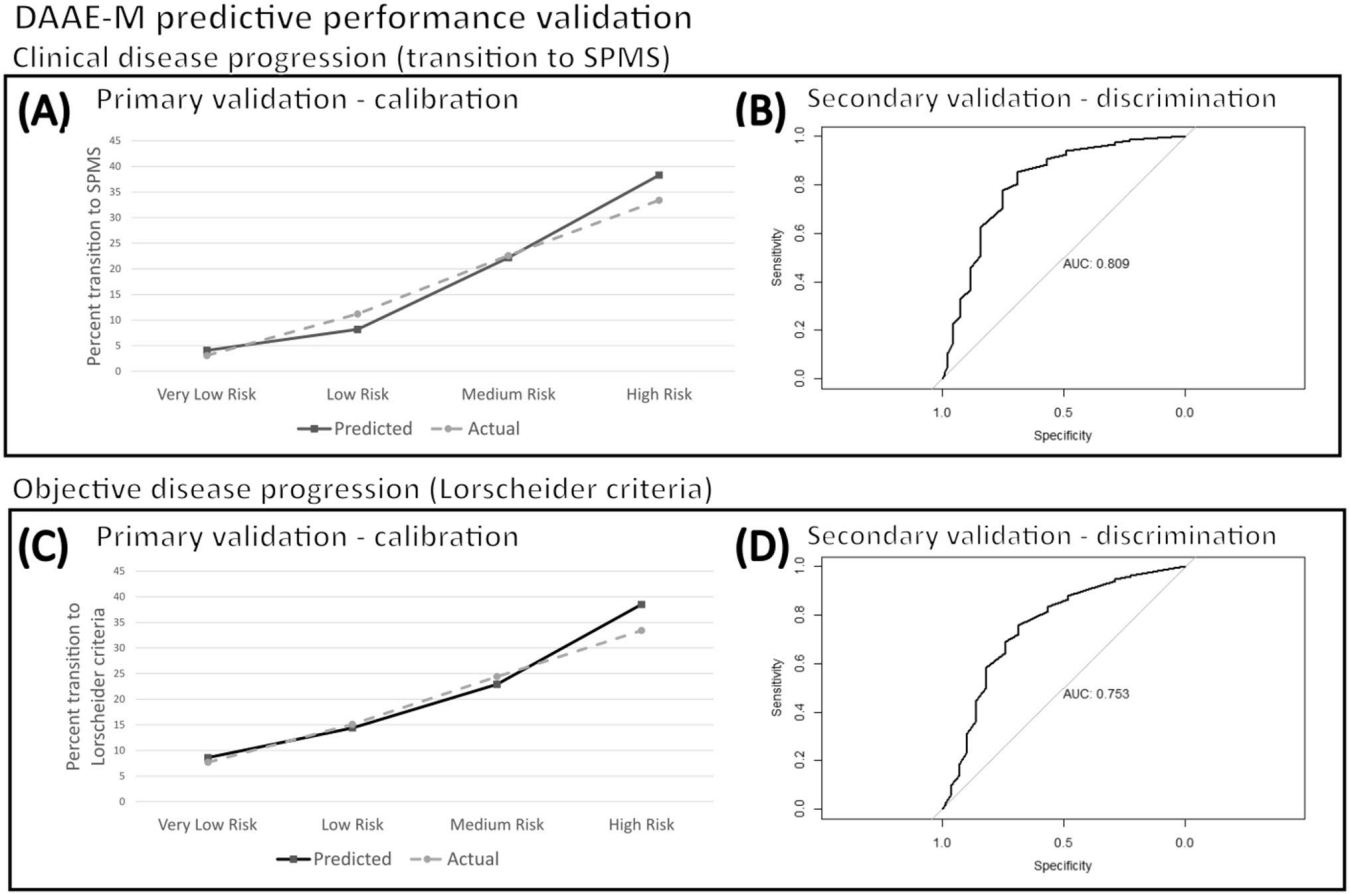
Predictive performance (DAAE-M score). Primary calibration analyses for clinically defined disease progression (**A**); the clinician-determined transition to SPMS) and secondary validation with discriminative testing (**B**). Primary calibration analyses for objectively-defined disease progression (**C**; the Lorscheider MSBase criteria for late-stage confirmed progression independent of relapse) and secondary discriminative testing (**D**) over the 5-year observation window. Abbreviations: *AUC* area under the receiver operating curve

Here, we aimed to validate the DAAE-M risk-stratification tool in a heterogeneous multi-center dataset according to clinically-defined disease progression (Transition to SPMS; Fig. 1B; Supplementary Table B). For details, see Methods and for score calculation, see supplemental materials. Using the DAAE-M score, PwMS were stratified into risk groups of very-low (*n* = 15,614), low (n = 13,904), medium (*n* = 2975), and high (*n* = 2017). The proportion of PwMS exhibiting clinical disease progression increased proportionally across these groups: very-low = 3.1%, low = 11.2%, medium = 22.6%, and high = 33.4%. These proportions per risk group were statistically significantly different from each other (*p* < 0.001), indicating the DAAE-M score successfully generated distinct escalating stratified risk groups. As well, in calibration testing, actual event proportions were consistent with predicted risk for the very-low, medium, and high-risk groups (*p* > 0.05), though different for the low-risk group (*p* = 0.002), Brier score = 0.08 (Fig. 2A).

Secondary validation discriminative testing using SMOTE + TK balanced case–control data (case *n* = 10,173; control *n* = 10,173) resulted in an AUROC = 0.809 (95% CI 0.803–0.815) (Fig. 2B), and an accuracy of 75.7% (95% CI 75.0–76.3).

#### Objective disease progression (Lorscheider criteria)

Here, we aimed to evaluate DAAE-M score performance in relation to objective disease progression criteria, namely late-stage confirmed disability progression independent of relapse using the MSBase-Lorscheider criteria [8]. Within PwMS meeting criteria for this analysis (*n* = 23,102), all PwMS (100%) were RRMS at baseline (per criteria). From these, 4856 (21.0%) exhibited objective disease progression over 5 years. This was significantly higher (*p* < 0.001) than the proportion exhibiting clinically-defined disease progression (9.8%).

DAAE-M score risk group stratification cutoffs were adapted for objective disease progression criteria as follows: very-low (0–1 points, *n* = 5586 [24.2%]), low (2–3 points, *n* = 6000 [25.9%]), medium (4–6 points, *n* = 6116 [26.5%]), and high (7–12 points, *n* = 5401[23.4%]). With this schema, the proportion of PwMS exhibiting objective disease progression increased stepwise across DAAE-M score stratified risk-groups: very-low = 8.6% (95% CI 7.7–9.2), low = 14.5(95% CI 13.6–15.4), medium = 23.3% (95% CI 22.2–24.3), high = 38.8% (95% CI 37.5–40.1). In an 80–20 randomized split, risks were well-calibrated across development [very-low = 8.6%(95% CI 7.7–9.2), low = 14.4 (95% CI 13.4–15.4), medium = 22.9% (95% CI 21.8–24.1), high = 38.5% (95% CI 37.1–40.0)] and validation [very-low = 7.7% (95% CI 6.2–9.4), low = 15.1(95% CI 13.1–17.2), medium = 24.4%(95% CI 21.9–26.9), high = 39.7% (95% CI 36.8–42.6)] data; Brier score = 0.16 (Fig. 2C).

Results pertaining to DAAE-M risk group stratification according to DMT use are presented in Table 3. In the majority-use high-efficacy treatment group, relative to the low-efficacy treatment group, risk was significantly reduced in particular for the low- (RR = 0.34 (95% CI 0.12–0.97), *p* = 0.043), and high risk groups (RR = 0.49 [95% CI 0.35–0.69], *p* < 0.001). In the no-treatment group relative to the majority low-efficacy treatment group, risk was significantly higher, in particular for the very-low- (RR = 3.4 [95% CI 1.5–7.5], *p* = 0.004) and medium risk groups (RR = 1.4 [95% CI 1.1–1.9], *p* = 0.049). Differences were non-significant for the remaining comparisons.

**Table 3:** Objective disease progression (Lorscheider criteria for late-stage confirmed progression independent of relapse) risk stratification, accounting for DMT use over the five-year observation window (DAAE-M score)

DAAE-M score	0–2	3–7	8–9	≥ 10
Risk group	Very low	Low	Medium	High
Risk of transition % (95% CI)				
Unspecified therapy	8.4% (7.7–9.2)	14.5% (13.6–15.4)	23.5% (22.2–24.3)	38.8% (37.5–40.1)
Majority DMT*				
Low-efficacy DMT	1.9% (0.8–3.8)	6.1% (4.1–8.5)	10.6% (8.1–13.6)	27.8% (23.5–32.4)
High-efficacy DMT	2.0% (0.4–5.8)	2.1% (0.6–5.3)	7.8% (5.0–11.6)	13.8% (9.9–18.5)
No DMT	6.3% (4.1–9.4)	6.1% (4.1–8.9)	14.8% (11.8–18.3)	32.4% (27.9–37.1)
Baseline DMT				
Low-efficacy DMT	4.9% (2.8–8.0)	9.5% (6.8–12.7)	12.3% (9.5–15.5)	26.1% (22.3–30.1)
High-efficacy DMT	7.0% (3.4–12.5)	4.7% (2.3–8.5)	11.2% (7.9–15.3)	21.9% (17.8–26.6)
No DMT	3.2% (1.6–5.6)	4.9% (3.0–7.6)	12.7% (9.9–15.9)	24.9% (21.1–29.0)

*CI* confidence interval, *DMT* disease-modifying therapy

^*^Majority DMT use is defined according to majority (> 50%) use of the five-year observation period. Unspecified therapy relates to data from the full study sample, regardless of DMT history

^**^Insufficient data was available for risk stratification in people who switched DMT class over the observation period

According to baseline DMT use, the high-efficacy therapy group had reduced risk in comparison with the low-efficacy therapy, in particular for the low-risk group (RR = 0.49 [95% CI 0.25–0.98], *p* = 0.043). Differences were non-significant for the remaining comparisons.

Secondary discriminative validation in SMOTE + TK balanced case–control data (case *n* = 9712; control *n* = 9712) resulted in an AUROC = 0.753 (95% CI 0.744–0.757) (Fig. 2D), and accuracy of 71.1% (95% CI 70.3–72.5).

## Predictive model optimizing dynamic estimation of risk and mitigation of immortal time bias (ELIE)

The model for optimization of complex treatment histories, immortal time-bias mitigation, and dynamic landmark-based estimation of risk is referred to as ELIE (Empiric Landmark-based Individualized Estimation of MS progression risk).

### Clinical disease progression (transition to SPMS)

**Fig. 3:**
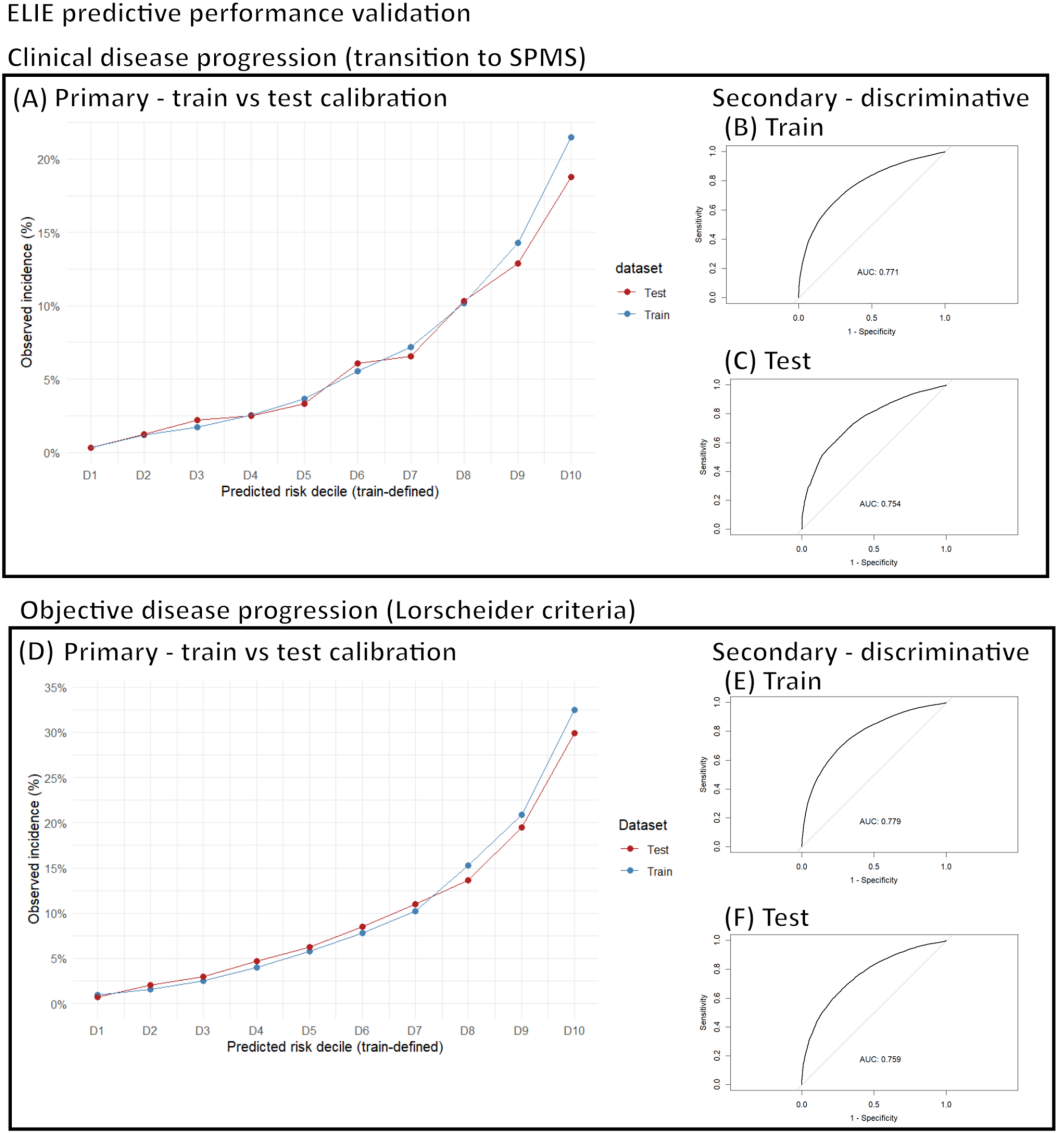
ELIE (empirical landmark-based individualized estimation of MS progression risk) predictive performance validation over a 5-year time-horizon. Primary calibration validation according to clinical disease progression (transition to SPMS) over a 5-year time-horizon (**A**) and secondary discriminative testing (**B**, **C**). Primary calibration validation according to objective disease progression (Lorscheider criteria) over a 5-year time-horizon (**D**) and secondary discriminative testing (**E**, **F**)

Risk of clinical disease progression (Fig. 3A) increased stepwise across ELIE stratified risk deciles in the training (0.3%, 1.2%, 1.7%, 2.5%, 3.7%, 5.5%, 7.2%, 10.2%, 14.3%, 21.5%) and test set (0.3%, 1.2%, 2.2%, 2.5%, 3.3%, 6.1%, 6.6%, 10.3%, 12.9%, 18.8%); Brier score = 0.057. The incidence values between train and test data for each risk decile were not statistically different from each other (*p* > 0.05) except for decile-10 (Fig. 3A; *p* < 0.001). For more details, see Supplementary Table C.

Secondary discriminative validation (Fig. 3B, C) resulted in an AUROC = 0.768 and an accuracy of 73.1% in the training data and an AUROC = 0.767 and an accuracy of 72.6% in the test data.

### Objective disease progression (Lorscheider criteria)

**Fig. 4:**
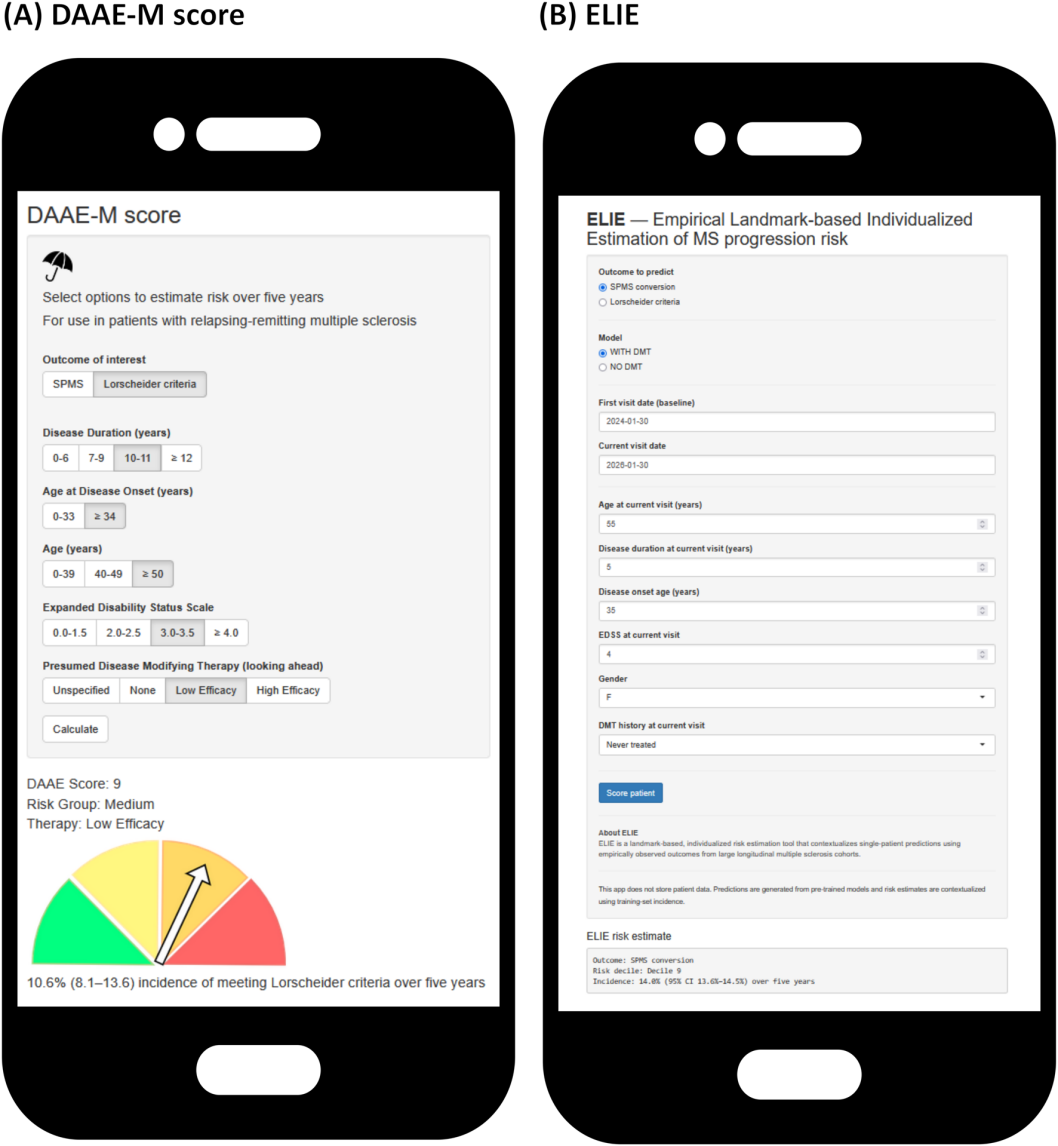
Sample use of the DAAE-M score (**A**) and ELIE (**B**) online web apps. Abbreviations: *CI* confidence interval; *DAAE-M* disease duration, age, age at onset, Expanded Disability Status Scale, disease-modifying therapy; *ELIE* empirical landmark-based individualized estimation of MS progression risk

Risk of objective disease progression (Fig. 3D) increased stepwise across ELIE stratified risk deciles in the training (0.9%, 1.6%, 2.5%, 4.0%, 5.8%, 7.8%, 10.2%, 15.3%, 20.9%, 32.5%) and the test set (0.7%, 2.0%, 3. 0%, 4.7%, 6.2%, 8.5%, 11.0%, 13.6%, 19.5%, 29.9%); Brier score = 0.084. These incidence values between the train and test data for each risk decile were not statistically different from each other (Fig. 4D; *p* > 0.05) except for decile-10 (*p* < 0.001). For more details, see Supplementary Table D.

Secondary discriminative validation (Fig. 3E-3F) resulted in an AUROC = 0.779, and accuracy of 76.5% in the training data and AUROC = 0.759 and accuracy of 74.9% in the test data.

## Open science and public tools

With the results from this study, we have generated the DAAE-M score and ELIE. A sample of the free online apps is presented in Fig. 4 and is available online (https://bit.ly/ms_public_tools). Printer-friendly versions of the tools are available in the supplemental materials.

## Discussion

To promote personalized patient care, we developed two models for individualized predictions of disease progression in PwMS; each has distinct trade-offs depending on what was optimized in the models. Herein, we present the DAAE-M score (Disease duration, Age, Age at disease onset, EDSS, Disease modifying therapy) and ELIE (Empiric Landmark-based Individualized Estimation of MS progression risk), which both integrate DMT information and multiple definitions of disease progression into probabilistic risk prediction.

### Principal findings

The DAAE-M score was developed in accordance with WHO guidelines for clinically-directed decision-support model development [4, 5]. Thus, this system was optimized for user operability (30 seconds to use), model transparency, and software-neutral interoperability to promote integration in clinics and electronic medical systems. This system was also optimized for mitigation of indication bias. As expected, in these analyses PwMS were stratified into groups of escalating risk concordantly with higher scores. As well, PwMS receiving high-efficacy DMT over the majority of the observation period exhibited half the risk relative to those receiving low-efficacy DMT across the risk groups (Fig. 1A, Table 2). Risks were similar when comparing patients in relation to baseline therapy.

Next, we aimed to enhance the generalizability of the scoring system by validating it in a large heterogeneous multi-center dataset. The DAAE-M score demonstrated strong performance in primary validation. Specifically, higher scores were associated with a higher actual likelihood of clinically defined disease progression (Fig. 1B), where event proportions were commensurate with risk predictions (Fig. 2A).

Then, we aimed to validate the performance of this tool against objective disease progression criteria. In this analysis, the DAAE-M score performed well stratifying PwMS according to risk of transition to the MSBase–Lorscheider criteria for late-stage confirmed progression independent of relapse activity, with escalating risk in association with higher risk groups and good performance in primary calibration analyses (Fig. 2C) and secondary discriminative analyses (Fig. 2D). The DAAE-M score public web app and development reference code are freely available https://bit.ly/ms_public_tools

Finally, we also developed an alternate predictive system, ELIE. This system, optimized for dynamic estimation of risk and mitigation of immortal time bias, performed well according to primary calibration analyses (actual event proportions commensurate with predictions) and according to secondary discriminative analyses. The dynamic landmark-based predictions allowed for more complex modeling—which also promoted greater potential for fine-tuned risk stratification schema and improved Brier scores (Fig. 3). However, this model is more opaque in its predictions, takes longer to use, and the model could not be stored in a software-neutral algorithm. Nonetheless, we have also made this model freely available on a public web app: https://bit.ly/ms_public_tools

### Relevance and comparison with external literature

These findings affirm the clinical risk stratification performance of new tools across clinically heterogeneous multi-center data, thus increasing generalizability for making prognostic estimates. Both the DAAE-M score and ELIE use similar predictors to those described in clinical trials [19]. They are inspired by previously developed tools [20, 21], they and function in a similar manner to known risk-stratification tools used in neurologic [22], and non-neurologic [23] populations. The DAAE-M score specifically matches the format of individual patient risk reporting using ordinalized scoring and software-neutral reference tables.

This tool has performed well in calibration analyses of risk predictions across several clinical environments [3, 24, 25]. The present work reflects a significant milestone, as it represents a large international validation, reinforcing generalizability across diverse clinical settings and countries. Importantly, this work also incorporates DMT history into new tools for providing patient risk estimates and also extends beyond subjective clinical judgment-based end-points by incorporating objective outcomes, thereby underscoring performance not only across new populations but also against standardized measures.

Additionally, the integration of DMT efficacy into DAAE-M score risk reports now allows for patients and physicians to simulate risk estimates specific to DMT types, according to baseline or majority use. This improves utility for personalized patient care associated with preliminary therapeutic choices or decisions to change therapies in high-risk patients. Importantly, our use of propensity-score matching for the model development mitigated the effects of indication bias [26] which can at times result in retrospective effects that paradoxically oppose [27] observed effects of treatment on clinical progression in randomized clinical trials [28]. With this methodology, our findings showing lower risk of clinical disease progression in PwMS receiving high-efficacy DMT are consistent with existing literature. For instance, in one study by Brown et al. [29], high-efficacy DMT was associated with a hazard-ratio of 0.66 in relation to risk of transition to SPMS for PwMS receiving low-efficacy DMT. This is similar to the hazard ratios reported in the present work, indicating that PwMS receiving high-efficacy DMT had hazard ratios ranging from 0.42 to 0.59 relative to low-efficacy DMT treated PwMS across the DAAE-M score stratified risk groups.

The modeling systems presented also performed well in predicting risk of transition to objectively defined disease progression using the MSBase-Lorscheider criteria. This is important because clinical definitions of disease progression can be heterogeneous between physicians and research centers [30]. Additionally, low sensitivity of testing and non-standardized definitions may result in diagnostic delay of SPMS [6], making use of objective criteria particularly important for identifying PwMS in transitional phases [31].

Finally, the findings presented, in addition to the previously described high ratings of the previous risk score on the System Usability Scale (90.3 out of 100) from physicians in over 20 countries [3], support its utility and feasibility for use in clinical environments. This tool can be used in personalized clinical care such as for supporting decisions whether to start or stop certain medicines [9], or deciding whether rehabilitation or other behavioral changes are appropriate [13, 32]. Similarly, the DAAE-M score can open conversations between PwMS and physicians about goals of care [33] and the desired path for care in balance with known risks.

In comparison with the DAAE-M score, ELIE’s strong performance in primary calibration and secondary discriminative testing is also worth consideration. This system has a number of strengths. Foremost, this tool provides dynamic risk estimates along the course of patient observation histories—accounting for observation time within its predictions. Along its dynamic handling of immortal time bias, ELIE also supports more complex retrospective treatment histories and treatment timing in its predictions and avoids selection biases in which certain treatment histories are excluded [9, 15]. Likewise, early success of the Barcelona risk score [34] and the SP-RiSc [21] illustrates the potential for alternate more complex predictive systems. However, these strengths of the ELIE model come with trade-offs, namely that it is less transparent, requires more time to be used, and requires specific software (e.g. r with appropriate machine-learning libraries) for integration into distinct international medical systems. Illustrated sample use of both DAAE-M and ELIE is presented in Fig. 4.

## Limitations and future directions

One key limitation of this work is that all results are derived from adult PwMS, thus neglecting applications in pediatric populations. Future work is needed to fill this gap. Additionally, neither of the presented tools incorporates MRI data. This, and the low proportion of patients in the present sample receiving high-efficacy therapy (7.2%) should be addressed in future work. Similarly, given the critical role of therapy timing [35], it is important to weigh trade-offs of inclusion of such information against priorities such as transparency and interoperability of models. As brain and spine MRI are shown to predict MS disease progression [36, 37], with the inclusion of MRI data in future models it will be vital to consider the feasibility of translation of MRI metrics depending on their complexity [38]. For instance, lesion counts and linear brain atrophy measures have low granularity, but are accessible in clinical environments, making translation more feasible [37]. Likewise, time required for tool use and availability of data (e.g. MRI scan type) must be heavily considered. For instance, FLAIR images are widely available clinically and improve predictions of future disease worsening [39], making them more ideal targets for clinical translation. The Barcelona risk score [34] and the SP-RiSc [21] illustrate the potential for MRI incorporation into similar models—demonstrating a step towards such approaches in future clinical practice.

Importantly, neither DAAE-M nor ELIE considers subtle forms of disability not well-captured by the EDSS, such as fatigue and cognition [40], nor subtle disability accrual independent of relapse in early MS stages [41]. It is important for future work to consider these limitations to improve predictive systems and to enable a wider range of prognostic clinical tools for PwMS.

## Conclusion

To promote personalized patient care, we developed two well-calibrated machine-learning-based tools for individualized 5-year predictions of clinically and objectively defined MS progression. The DAAE-M score was optimized according to WHO guidelines for transparency, software-neutral interoperability, and for mitigation of indication bias. In comparison, ELIE was optimized for dynamic modeling, complex treatment histories, and mitigation of immortal-time bias. Both systems performed well in primary calibration analyses, with predicted risk closely matching actual incidence, albeit with distinct trade-offs per system depending on what was optimized for in development. These results support the use of such risk stratification systems for identifying target PwMS for relevant secondary prevention and restorative treatment strategies. Future work should incorporate MRI and neuropsychological data to further enhance use for prognostication and personalized patient care.

## Supplementary Information

Below is the link to the electronic supplementary material.

Supplementary file1 (DOCX 19 KB)

Supplementary file2 (DOCX 31 KB)

Supplementary file3 (DOCX 32 KB)

## Data Availability

Anonymized data use for the present analyses are available upon reasonable request from the MSBase patient regestry
