# Supplementary material for: Predicting disease progression in multiple sclerosis with clinically accessible information and technology
Source: J Neurol. 2026 Apr 19;273(5):281. doi: 10.1007/s00415-026-13802-4 (PMC13092528; doi:10.1007/s00415-026-13802-4)
Supplement: Supplementary file 1 — Supplementary file1 (DOCX 19 KB) [file 415_2026_13802_MOESM1_ESM.docx]

|  | **DAAE-M score** | 0-2 | 3-7 | 8-9 | ≥10 |
| --- | --- | --- | --- | --- | --- |
|  | **Risk group** | **Very low** | **Low** | **Medium** | **High** |
| Sample (n)** | **Unspecified therapy** | 15,614 | 13,904 | 2,975 | 2,017 |
|  | **Low-efficacy DMT** | 974 | 1,052 | 244 | 212 |
|  | **High-efficacy DMT** | 957 | 1,063 | 297 | 165 |
|  | **No DMT** | 1,018 | 1,136 | 208 | 120 |
|  | **Switch DMT class** | 1,724 | 757 | 1 | 0 |

**Supplementary Table A**. Sample sizes for risk stratification, accounting for DMT use* over the five-year observation window (DAAE-M)

DMT=disease modifying therapy

*DMT history defined according to majority (>50%) use of the five-year observation period. Unspecified therapy relates to data from the full study sample, regardless of DMT history.

**sample sizes of DMT groups derived from propensity score matched analyses

**Supplementary Table B**. Percent clinical disease progression over five years for stratified risk groups (DAAE-M score)

|  | **Previous Investigations**  n=2,186 | | **MSBase Validation**  n=34,510 | |
| --- | --- | --- | --- | --- |
| **Risk Group** | n patients  (%) | % conversion  (95% CI) | n patients  (%) | % conversion  (95% CI) |
| Very Low (0-2) | 493  (22.8%) | 4.1  (2.5-6.2) | 15,614  (45.2%) | 3.1  (2.9-3.4) |
| Low (3-7) | 1,060  (49.1%) | 8.2  (6.6-10.0) | 13,904  (40.3%) | 11.2  (10.7-11.8) |
| Medium (8-9) | 347  (16.1%) | 22.2  (17.9-26.9) | 2,975  (8.6%) | 22.6  (21.1-24.1) |
| High (≥10) | 256  (11.9%) | 38.3  (32.3-44.5) | 2,017  (5.8%) | 33.4  (31.0-35.1) |

**Supplementary Table C**. Clinical disease progression (transition to SPMS) for empirical landmark-based individualized estimation of MS progression risk (ELIE). Train vs test incidence across risk deciles.

| Risk group | n train | Events train | n test | Events test | Incidence train (%) | Incidence test (%) | 95% CI train | 95% CI test |
| --- | --- | --- | --- | --- | --- | --- | --- | --- |
| Decile 1 | 26846 | 85 | 6570 | 22 | 0.3 | 0.3 | 0.3-0.4 | 0.2-0.5 |
| Decile 2 | 26845 | 314 | 6602 | 81 | 1.2 | 1.2 | 1.0-1.3 | 1.0-1.5 |
| Decile 3 | 26845 | 461 | 6885 | 152 | 1.7 | 2.2 | 1.6-1.9 | 1.9-2.6 |
| Decile 4 | 26845 | 677 | 6913 | 172 | 2.5 | 2.5 | 2.3-2.7 | 2.1-2.9 |
| Decile 5 | 26845 | 982 | 6696 | 221 | 3.7 | 3.3 | 3.4-3.9 | 2.9-3.8 |
| Decile 6 | 26845 | 1485 | 7111 | 433 | 5.5 | 6.1 | 5.3-5.8 | 5.5-6.7 |
| Decile 7 | 26845 | 1926 | 7049 | 462 | 7.2 | 6.6 | 6.9-7.5 | 6.0-7.2 |
| Decile 8 | 26845 | 2734 | 6559 | 676 | 10.2 | 10.3 | 9.8-10.6 | 9.6-11.1 |
| Decile 9 | 26845 | 3837 | 6407 | 825 | 14.3 | 12.9 | 13.9-14.7 | 12.1-13.7 |
| Decile 10 | 26846 | 5771 | 6329 | 1189 | 21.5 | 18.8 | 21.0-22.0 | 17.8-19.8 |

CI=confidence interval

**Supplementary Table D**. Objective disease progression (Lorscheider criteria) for empirical landmark-based individualized estimation of MS progression risk (ELIE). Train vs test incidence across risk deciles.

| Risk group | n train | Events train | n test | Events test | Incidence train (%) | Incidence test (%) | 95% CI train | 95% CI test |
| --- | --- | --- | --- | --- | --- | --- | --- | --- |
| Decile 1 | 25973 | 238 | 6298 | 47 | 0.9 | 0.7 | 0.8-1.0 | 0.5-1.0 |
| Decile 2 | 25972 | 413 | 6259 | 123 | 1.6 | 2 | 1.4-1.7 | 1.6-2.3 |
| Decile 3 | 25972 | 658 | 6090 | 180 | 2.5 | 3 | 2.3-2.7 | 2.5-3.4 |
| Decile 4 | 25972 | 1039 | 6212 | 289 | 4 | 4.7 | 3.8-4.2 | 4.1-5.2 |
| Decile 5 | 25973 | 1517 | 6286 | 388 | 5.8 | 6.2 | 5.6-6.1 | 5.6-6.8 |
| Decile 6 | 25972 | 2023 | 6735 | 573 | 7.8 | 8.5 | 7.5-8.1 | 7.9-9.2 |
| Decile 7 | 25972 | 2655 | 6796 | 747 | 10.2 | 11 | 9.9-10.6 | 10.3-11.8 |
| Decile 8 | 25972 | 3961 | 6712 | 910 | 15.3 | 13.6 | 14.8-15.7 | 12.7-14.4 |
| Decile 9 | 25972 | 5423 | 6446 | 1255 | 20.9 | 19.5 | 20.4-21.4 | 18.5-20.5 |
| Decile 10 | 25973 | 8443 | 7098 | 2120 | 32.5 | 29.9 | 31.9-33.1 | 28.8-30.9 |

CI=confidence interval
