# Supplementary material for: Predicting disease progression in multiple sclerosis with clinically accessible information and technology
Source: J Neurol. 2026 Apr 19;273(5):281. doi: 10.1007/s00415-026-13802-4 (PMC13092528; doi:10.1007/s00415-026-13802-4)
Supplement: Supplementary file 2 — Supplementary file2 (DOCX 31 KB) [file 415_2026_13802_MOESM2_ESM.docx]

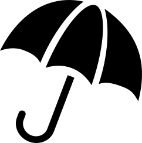
**DAAE-M score**. Score calculation and percent risk of transition to secondary progressive multiple sclerosis over five years. For use with relapsing-remitting multiple sclerosis patients.

To calculate the **DAAE-M score**, determine the points scored for each factor in **Table** **(A)** and calculate the sum. Reference the **DAAE-M score** with risk group in **Table** **(B)**.

**(A)**

| **DAAE-M Score** | | | | | **Points** |
| --- | --- | --- | --- | --- | --- |
| **D**isease **D**uration | 1-6 | 7-9 | 10-11 | ≥ 12 |  |
|  | **+0** | **+1** | **+2** | **+3** |  |
| **A**ge at Onset | 0-33 | ≥ 34 | - | - |  |
|  | **+0** | **+1** |  |  |  |
| **A**ge | 0-39 | 40-49 | ≥ 50 | - |  |
|  | **+0** | **+1** | **+2** |  |  |
| **E**DSS | 0-1.5 | 2.0-2.5 | 3.0-3.5 | ≥ 4.0 |  |
|  | **+0** | **+2** | **+4** | **+6** |  |
| **Sum for Final Score** | | | | |  |

**(B)**

|  | **DAAE-M Score** | 0-2 | 3-7 | 8-9 | ≥10 |
| --- | --- | --- | --- | --- | --- |
|  | **Risk Group** | **Very Low** | **Low** | **Medium** | **High** |
| **Risk of Transition**  % (95% CI) | **Unspecified Therapy** | 3.1%  (2.9-3.4) | 11.2%  (10.7-11.8) | 22.6%  (21.1-24.1) | 33.4%  (31.0-35.1) |
|  | **Low-Efficacy DMT*** | 3.3%  (2.3-4.6) | 10.3%  (8.5-12.3) | 18.4%  (13.8-23.8) | 40.6%  (33.9-47.5) |
|  | **High-Efficacy DMT*** | 1.8%  (1.0-2.8) | 6.6%  (5.2-8.2) | 13.5%  (9.8-17.9) | 15.2%  (10.1-21.5) |
|  | **No DMT** | 5.2%  (3.9-6.8) | 16.7%  (14.6-19.0) | 26.9%  (21.0-33.5) | 35.0%  (26.5-44.2) |

**Abbreviations**: EDSS=expanded disability status scale; DMT=disease modifying therapy; CI=confidence interval

*DMT is presumed to be used over the majority (>50%) of the five-year period. Unspecified therapy relates to estimated risks without accounting for DMT.

**Background**: The **DAAE-M Score** is named from factors included in the predictive model: **D**isease duration, **A**ge at disease onset, **A**ge, **E**xpanded Disability Status Scale, and disease **m**odifying therapy. This work was supported by the European Consortium for Treatment and Research in Multiple Sclerosis. Unspecified therapy estimates are based on international combined data from the United States (Jacobs Multiple Sclerosis Center, n=1,309), Netherlands (Multiple Sclerosis Center Amsterdam, n=877), and the MSBase multi-center international dataset (n=34,510). DMT efficacy DMT risk estimates are based on a propensity-score-matched data subset from the MSBase registry (n=9,547).
