# Supplementary material for: Predicting disease progression in multiple sclerosis with clinically accessible information and technology
Source: J Neurol. 2026 Apr 19;273(5):281. doi: 10.1007/s00415-026-13802-4 (PMC13092528; doi:10.1007/s00415-026-13802-4)
Supplement: Supplementary file 3 — Supplementary file3 (DOCX 32 KB) [file 415_2026_13802_MOESM3_ESM.docx]

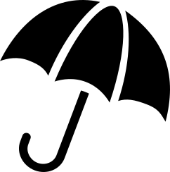
**DAAE-M score**. Score calculation and percent risk of transition to objective Lorscheider-MSBase disease progression criteria over five years. Intended for use with relapsing-remitting multiple sclerosis patients.

To calculate the **DAAE-M score**, determine the points scored for each factor in **Table** **(A)**. The total sum of these points is the **DAAE-M score**. Reference the **DAAE Score-M** with risk group and percent risk of conversion in **Table** **(B)**.

**(A)**

| **DAAE-M Score** | | | | | **Points** |
| --- | --- | --- | --- | --- | --- |
| **D**isease **D**uration | 1-6 | 7-9 | 10-11 | ≥ 12 |  |
|  | **+0** | **+1** | **+2** | **+3** |  |
| **A**ge at Onset | 0-33 | ≥ 34 | - | - |  |
|  | **+0** | **+1** |  |  |  |
| **A**ge | 0-39 | 40-49 | ≥ 50 | - |  |
|  | **+0** | **+1** | **+2** |  |  |
| **E**DSS | 0-1.5 | 2.0-2.5 | 3.0-3.5 | ≥ 4.0 |  |
|  | **+0** | **+2** | **+4** | **+6** |  |
| **Sum for Final Score** | | | | |  |

**(B)**

|  | **DAAE-M Score** | 0-1 | 2-3 | 4-6 | 7-12 |
| --- | --- | --- | --- | --- | --- |
|  | **Risk Group** | **Very Low** | **Low** | **Medium** | **High** |
| **Risk of Transition**  % (95% CI) | **Unspecified Therapy** | 8.4%  (7.7-9.2) | 14.5%  (13.6-15.4) | 23.3%  (22.2-24.3) | 38.8%  (37.5-40.1) |
|  | **Low-Efficacy DMT*** | 1.9%  (0.8-3.8) | 6.1%  (4.1-8.5) | 10.6%  (8.1-13.6) | 27.8%  (23.5-32.4) |
|  | **High-Efficacy DMT*** | 2.0%  (0.4-5.8) | 2.1%  (0.6-5.3) | 7.8%  (5.0-11.6) | 13.8%  (9.9-18.5) |
|  | **No DMT** | 6.3%  (4.1-9.4) | 6.1%  (4.1-8.9) | 14.8%  (11.8-18.3) | 32.4%  (27.9-37.1) |

**Abbreviations**: EDSS=expanded disability status scale; DMT=disease modifying therapy; CI=confidence interval

*DMT is presumed to be used over the majority (>50%) of the five-year period

**Background**: The **DAAE-M Score** is named from factors included in the predictive model: **D**isease duration, **A**ge at disease onset, **A**ge, **E**xpanded Disability Status Scale, and disease **m**odifying therapy. This work was supported by the European Consortium for Treatment and Research in Multiple Sclerosis. Unspecified therapy estimates are based on international combined data from the United States (Jacobs Multiple Sclerosis Center, n=1,309), Netherlands (Multiple Sclerosis Center Amsterdam, n=877), and the MSBase multi-center international dataset (n=34,510).
